# Autonomy under pressure: a scoping review of social egg freezing in the bottom quintile of the gender gap index

**DOI:** 10.1186/s12910-025-01353-8

**Published:** 2026-01-05

**Authors:** Shizuko Takahashi, Zakiyya Muhyideen Abdullah, Mathavi Senguttuvan, Henry Dobson, Mohammed Ghaly

**Affiliations:** 4 Centre for Biomedical Ethics, Yong Loo Lin School of Medicine, National University of Singapore, Block MD11, Clinical Research Centre, #02-03, 10 Medical Drive , Singapore, Singapore 117597 Singapore; 1 Department of Biomedical Ethics, The University of Tokyo, Graduate School of Medicine, Tokyo, Japan; 2 Department of Bioethics & Humanities, University of Washington, School of Medicine, Seattle, WA USA; 3 Research Center for Islamic Legislation & Ethics, College of Islamic Studies, College of Health and Life Sciences, Hamad Bin Khalifa University, Doha, Qatar

**Keywords:** Oocyte cryopreservation, Reproductive autonomy, Gender, Social norms, Fertility preservation, Islamic law, Pronatalism, Reproductive ethics, Infertility

## Abstract

**Background:**

Social Egg Freezing (SEF), often promoted in Western contexts as a means to enhance reproductive autonomy, has seen varied uptake in countries with wider gender disparities. In such settings, where reproductive decisions are shaped by strong societal norms, SEF raises ethical concerns about whether it enables autonomy or reinforces prevailing expectations.

**Objectives:**

This study examines how SEF is framed and regulated in countries ranking in the lowest quintile of the 2024 Gender Gap Index (GGI), where reproductive autonomy is emergent and SEF is increasingly promoted as a demographic intervention.

**Methods:**

A scoping review was conducted on SEF literature published between 2013 and 2025 across 29 countries in the lowest GGI quintile. English-language sources were retrieved from PubMed, while Japanese (J-Stage, Ichushi-web) and Arabic (Arabase [al-manẓūma], E-Marefa) were searched. Supplementary materials—including government policies, religious texts, and professional guidelines—were identified through Google Advanced Search, manual screening, and documents from the International Islamic Fiqh Academy (IIFA). Eligible sources included empirical and normative publications addressing SEF in the countries under review. Three reviewers screened and extracted data. Themes were developed iteratively.

**Results:**

Sixty-eight sources met inclusion criteria and were organized into five themes: (1) awareness and education; (2) sociocultural and religious values; (3) policy and legal frameworks; (4) clinical practices and outcomes; and (5) commercialization and autonomy. Most sources (73.9%) focused on themes (2)-(4), while awareness (12.2%) and commercialization (13.9%) received limited attention. In most Muslim-majority countries reviewed, religious frameworks restricted SEF to married women. Turkey is a notable exception, permitting SEF for single women though embryo transfer requires marriage. Only 23 of the 68 sources were empirical, and just four employed qualitative methods.

**Conclusion:**

Although SEF is often framed as empowering, this promise is context-dependent and frequently illusory. Cultural and structural constraints shape women’s choices, limiting the autonomy SEF claims to offer. Rather than imposing hegemonic neo-liberal assumptions, future policy and research should engage with local moral reasoning while examining how norms may reinforce inequality. Empirical studies are urgently needed to illuminate lived realities and improve policy development.

**Supplementary Information:**

The online version contains supplementary material available at 10.1186/s12910-025-01353-8.

## Background

Social egg freezing (SEF) has emerged as a focal point in discussions surrounding reproductive autonomy, particularly in Western contexts where it is often portrayed as an empowering technology. SEF supposedly enables women to preserve their fertility and maximize reproductive choices, whether due to the absence of a partner, medical considerations, career planning, or other personal circumstances, while maintaining the possibility of having genetically related children of their own in the future. Proponents argue that SEF enhances reproductive choice, allowing women to balance personal goals, career ambitions, and family planning [1]. Pennings claims that the line between “social” egg freezing and “medical” egg freezing is unclear, and SEF should therefore be allowed for any reason [1, 2]. High-profile corporate policies, such as those adopted by Apple and Facebook, underscore this framing by offering SEF as an employee benefit [3, 4]. However, these corporate initiatives have faced criticism for potentially pressuring women to postpone childbearing to align with workplace productivity norms rather than genuinely enhancing autonomy [5]. Critics argue that rather than addressing structural workplace barriers—such as inflexible work schedules, inadequate parental leave, or career penalties for motherhood—employer-sponsored SEF shifts the responsibility for managing fertility-work conflicts onto individual women while preserving organizational structures that disadvantage caregivers [6, 7].

Despite extensive bioethical discourse on SEF in Western countries, where extensive empirical studies have been conducted, significantly less attention has been paid to its adoption, motivations, and policy frameworks in non-Western contexts, particularly in countries with substantial gender disparities [8]. According to the World Economic Forum’s *Global Gender Gap Report 2023* [9], countries in the lower quintile—representing the bottom 20% in terms of gender parity—face deeply rooted structural barriers affecting women’s access to reproductive healthcare and their autonomy. Even in these contexts, fertility trends reveal that both the birthrate and the average age at which women have children are shifting [10]. Declining fertility rates, coupled with delayed childbearing, mirror global trends despite cultural differences and varying gender norms. For example, total fertility rates in Muslim-majority countries have declined substantially—from 6.0 children per woman in 1980 to 2.4 in 2020 in the Middle East and North Africa region—while the average maternal age at first birth has increased [10, 11]. In some of these countries, particularly Japan, SEF is increasingly promoted as a response to demographic challenges—specifically declining birth rates [12, 13] — rather than as a tool for individual empowerment. Governmental policies in some settings subsidize SEF to counter declining birthrates, raising ethical concerns about whether these interventions genuinely enhance women’s reproductive choices or subtly pressure them to fulfil societal roles.

Among the 29 countries in the lowest quintile of the 2024 Gender Gap Index, 23 are Muslim-majority nations. These countries collectively represent contexts where gender equality metrics are particularly low, yet where reproductive technologies including SEF are theoretically or practically available. Understanding SEF within these contexts requires urgent attention for understanding how Islamic jurisprudence, cultural norms, and gender inequality intersect. In the context of these Muslim-majority countries, SEF is both theoretically permissible in Islam and practically available in most settings. There are no explicit legal prohibitions against it, and the procedure is already offered at some healthcare institutions. However, practical concerns arise from the need to align the use of this technology with the religious and ethical values that shape both individual choices and the broader moral landscape of Muslim-majority societies. In some contexts, key considerations include ensuring that egg retrieval does not compromise a woman’s virginity—a matter of significant social and religious sensitivity in certain Muslim-majority countries [14]. If non-invasive methods are unavailable and the procedure risks affecting virginity, this should be properly documented by hospital authorities to protect the woman from future social harm, particularly in the context of marriage [15]. However, practices vary considerably across Muslim-majority countries; for instance, in Turkey’s more secular context, virginity concerns are primarily social rather than religious, and documentation remains optional based on patient choice rather than institutional requirement. Additionally, strict safeguards must be in place to ensure that the frozen eggs are used exclusively by the woman from whom they were collected and only within the bounds of a valid marriage. This is essential to preserve lineage integrity, in line with Islamic teachings, legal requirements, and prevailing social norms [15–17].

Following presentations on SEF policies and lived experiences in gender parity at the Feminist Approaches to Bioethics (FAB) 2024 Congress, the presenters collaborated with another attendee from India to explore broader patterns across bottom-quintile Gender Gap Index (GGI) countries.^1^ This collaboration prompted a comprehensive examination of SEF adoption, motivations, policies, and ethical considerations in these regions. Guided by the Joanna Briggs Institute (JBI) methodological guidance for scoping reviews, this study aims to map the extent and nature of existing literature, identifying key gaps and variations in SEF practices in countries with significant gender disparities [18].

We examined how SEF is framed in bottom-quintile GGI countries by mapping existing literature, analyzing its positioning within cultural and policy contexts, and identifying gaps needing further empirical and normative research.

## Methods

This study employed a scoping review methodology following the Joanna Briggs Institute’s (JBI) guidance for conducting scoping reviews. This approach is particularly suited to mapping broad and heterogeneous fields of research, identifying knowledge gaps, and clarifying key concepts [19]. Given the limited empirical data from many target countries, a broad approach to inclusion and exclusion criteria was adopted to capture diverse perspectives. While peer-reviewed literature was prioritized, some non-peer-reviewed sources were included due to the scarcity of research in certain regions and the difficulty in determining the peer-review status of some publications, particularly in non-English sources. The 29 countries in the bottom-quintile GGI reviewed in this study, along with their general attitudes toward assisted reproductive technology (ART) and prevailing religious contexts, are summarized in Table 1 (the full list of the 29 countries are in Additional File 1).

**Table 1:** Lower quintile of gender gap index countries 2024 with articles: ART, SEF Policies, and cultural context (Reference pertaining to Muslim Majority Countries is indicated in [9])

Rank (#articles)	Country	Predominant Religion	SEF Policy	Approximate SEF Cost (USD)	ART Law and Cultural Attitude
118 (23)	Japan	Shintoism/Buddhism (Secular)	Allowed; local government subsidies for egg freezing in some areas (up to $2600)^80^	$2,000–$3,000 per cycle (plus storage)	Guidelines-based; SEF and ART allowed, culturally cautious toward non-traditional families
123 (1)	Jordan	Islam	Permitted under ethical and religious guidelines; limited SEF discussion	$2,000–$4,000 per cycle	Permitted with restrictions; Islamic bioethics influence ART use
126 (5)	Saudi Arabia	Islam	Permitted for medical reasons; elective SEF restricted	$3,000–$5,000 per cycle	Strictly regulated; ART for married couples only under Islamic law
127 (7)	Turkey	Islam	Permitted for medical and SEF strictly regulated (diminished ovarian reserve, women above 38, and family history of premature menopause)^22^	$4,400 to $5,500 USD 81	Permitted under Ministry of Health guidance; SEF allowed for anticipated infertility; cultural tension remains
129 (10)	India	Hinduism	Permitted; regulated under ART Act (2021), SEF allowed for unmarried women	$1,000–$2,500 per cycle 82	ART Act (2021) governs use; socially stratified access, SEF legal
130 (2)	Qatar	Islam	Restricted; ART available to married couples, SEF uncommon	$3,000–$6,000 per cycle	Highly restricted; ART allowed for married couples only
133 (1)	Lebanon	Islam/Christianity	Permitted; SEF discussed, private clinic access	$4,000–5000 maximum per cycle^30^	Permitted; religiously diverse, private sector led, growing acceptance
135 (14)	Egypt	Islam	Permitted for medical reasons; social egg freezing not widely practiced	$1,500–$3,000 per cycle (private clinics)	Permitted under Islamic guidelines; social stigma limits openness
139 (1)	Algeria	Islam	Permitted under certain restrictions; SEF not regulated	$2,000–$3,500 per cycle	Permitted; Islamic law influences access and use
143 (2)	Iran (Islamic Republic of)	Islam	Permitted under Islamic bioethical framework; SEF limited	$1,200–$2,500 per cycle	Permitted under Islamic law; strong state ART support

### Search strategy and data sources

A comprehensive search strategy was developed to capture both empirical and normative literature. English-language sources were retrieved from PubMed and Google Scholar. Rayyan–a web-based software–was used to manage and sort English articles during the screening process.

To enhance regional representation, Japanese-language databases: J-Stage and Ichushi-web, and Arabic-language databases (Arabase [*al-manẓūma*] and E-Marefa), alongside publications from the International Islamic Fiqh Academy (IIFA), were thoroughly searched. Government policies, institutional guidelines, and relevant non-academic media content were identified through Google Search to supplement academic findings. Search terms included combinations of “social egg freezing,” “elective egg freezing,” “fertility preservation,” “elective oocyte cryopreservation,” and “planned oocyte cryopreservation.” For English-language searches, these terms were combined with specific country names to narrow the scope (e.g., “India,” “Turkey,” “Qatar”). Terms such as “cancer” and “onco-fertility” were excluded to focus on elective, non-medical contexts. The search was limited to literature published between January 1, 2013 and March 30, 2025. The year 2013 was selected as the starting point because it marked the removal of the ‘experimental’ label from oocyte cryopreservation by the American Society for Reproductive Medicine, after which SEF began to gain broader clinical acceptance and public attention globally [20].

### Selection criteria

Eligible sources included publications that addressed SEF in the specified countries, were published within the review period, and focused on women in relation to SEF. This encompassed empirical and normative studies, government policy/legaldocuments, professional guidelines, and religious texts directly influencing SEF discourse or practice.

Excluded were conference proceedings without full texts, inaccessible or duplicate published works; articles not primarily focused on SEF (e.g., medical egg freezing for cancer); studies using animal models; publications centred on male infertility or ovarian tissue cryopreservation; and those discussing unrelated reproductive interventions. Reviews or articles that did not substantively engage with the country of interest—despite the authors’ (of the paper) regional affiliation—were also excluded, unless contextual relevance could be clearly inferred.

### Screening and data extraction

Three independent reviewers screened titles and abstracts for relevance, followed by full-text evaluations of eligible studies. Discrepancies were resolved through discussion or consultation with a fourth and fifth reviewer (HD, MG). Extracted data included study objectives, methodologies, participant demographics, key findings, and alignment with the research question. Special attention was given to how SEF was framed within the respective country’s cultural and policy contexts.

### Researcher positionality

We acknowledge that our positioning as researchers influenced the design and interpretation of this study. The research team includes members from diverse backgrounds: ST (Japanese bioethicist in Singapore and a certified obstetrics and gynecologist who trained in Japan and the US), ZA (Islamic ethics scholar from Qatar), MS (legal scholar from India), HD (Australian bioethicist in Singapore), and MG (Islamic ethics scholar). This diversity enabled multilingual literature access and culturally informed interpretation, but also reflects our specific institutional and intellectual contexts. Our framing of SEF through gender inequality metrics and our emphasis on autonomy reflect particular bioethical commitments. We attempted to remain reflexive about how our frameworks might not fully capture local understandings, and engaged with literature that challenged our initial assumptions. Nevertheless, our interpretations remain partial and shaped by our positions within academic bioethics discourse.

### Data analysis

Themes were identified inductively through the review process rather than synthesized using a predefined framework. As studies were reviewed, recurring patterns across motivations, regulations, and sociocultural contexts of SEF were categorized into five thematic areas: [1] awareness and education; [2] socio-cultural, and religious perspectives; [3] legal, political, regulations, and religious governance structures; [4] clinical practices and outcomes; and [5] commercialization, and autonomy. Informal qualitative insights from regional consultations and presentations at the FAB 2024 Congress complemented the findings, providing contextual understanding. This approach enabled the mapping of emerging themes and highlighted how SEF is framed and practiced across these countries.

## Results

**Fig. 1:**
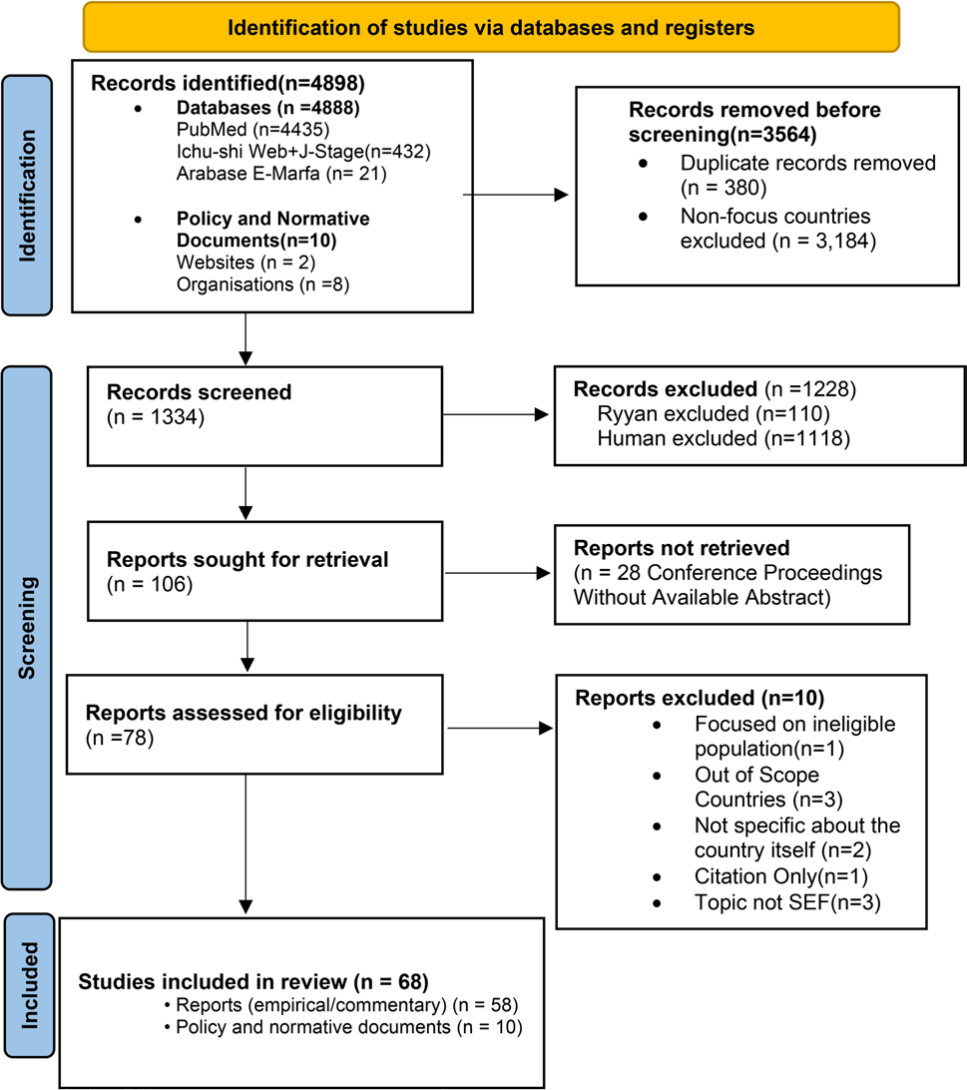
PRISMA flow diagram

A total of 4,894 records were identified: 4,888 from databases (PubMed, Ichushi-Web, J-Stage, Arabase, and *E-Marefa*) and 10 policy or normative documents from websites and organizations(e.g., Egypt’s *Dar Al-Ifta*, Tokyo Metropolitan Government, Qatar Foundation, and the Assisted Reproductive Technology Act from India). Using country-specific search terms, 3,184 records related to non-focus countries were excluded. After removing 380 duplicates, 1,334 records remained for title and abstract screening, of which 1,228 were excluded. A total of 106 records underwent full-text review. In the end, 68 sources were included in this scoping review. The PRISMA flow diagram is as shown in Fig. 1. The full reference for the 68 sources is as attached in Additional File 2.

### Distribution of the selected studies

Among the 29 bottom-quintile GGI countries included in the review, SEF-related publications were identified for Japan [23], Egypt [14], India [10], Turkey [7], Saudi Arabia [5], Multi-country Islamic perspectives^2^ [2], Iran [2], Qatar (2), Lebanon [1], Jordan [1], and Algeria [1]. Table 1 shows the countries and relative characteristics of the countries and their policies. Japan had the highest volume of publications, followed by Egypt, India, and Turkey, then Saudi Arabia. No articles were found specifically for Nepal, Comoros, Burkina Faso, Côte d’Ivoire, Sri Lanka, Nigeria, Fiji, Kuwait, Benin, Oman, Morocco, Niger, Democratic Republic of Congo, Mali, Guinea, Chad, Pakistan, Sudan, Maldives, and Nigeria, despite extensive searches.

Of the 68 sources included in this review, 28 were empirical studies, 40 were normative articles or regulatory documents retrieved from websites and organizational sources (Table 2). Only 4 articles were qualitative, 3 from Turkey [8, 21, 22] and 1 from Saudi Arabia [23]. The scoping review yielded articles that were categorized into five main thematic areas related to SEF: [1] Awareness, and education; [2] Socio-cultural and religious values; [3] Legal, political and religious governance; [4] Clinical practices and outcomes; and [5] Commercialization, and autonomy. The full labeling of the themes for the references is as shown in Additional File 3.

**Table 2:** Characteristics of included citations

	Peer-Reviewed Studies (*n* = 51) Number (%)	Non-Peer Reviewed + Gray Literature (*n* = 17) Number (%)
Country		
Japan	14 (27.4)	9(53)
Egypt	13 (25.4)	1(6)
India	9 (17.6)	1(6)
Turkey	7 (13.7)	
Saudi Arabia	3 (5.9)	2(12)
Iran	2 (3.8)	
Algeria	1 (2)	
Jordan	1 (2)	
Lebanon	1 (2)	
Qatar		2(12)
Muslim Majority Countries		2(12)
Empirical (*n* = 28 total)	24	4
• Case Report	1	
• Cross-Sectional Survey	12	4
• Retrospective Study of Patients	7	
• Qualitative	3	
• Mixed	1	
Normative(*n* = 40 total)	27	13

**Table 3:** Overall Instances of Themes. This table presents the frequency and percentage of each theme based on total coded instances (*n*=115), accounting for overlapping themes across 68 SEF literature documents

Theme	Count (*n*=115)	Percentage (%)
1. Awareness & Education	14	12.2
2. Sociocultural & Religious Values	28	24.3
3. Legal, Political & Religious Governance	27	23.5
4. Clinical Practices & Outcomes	30	26.1
5. Commercialization & Autonomy	16	13.9

**Fig. 2:**
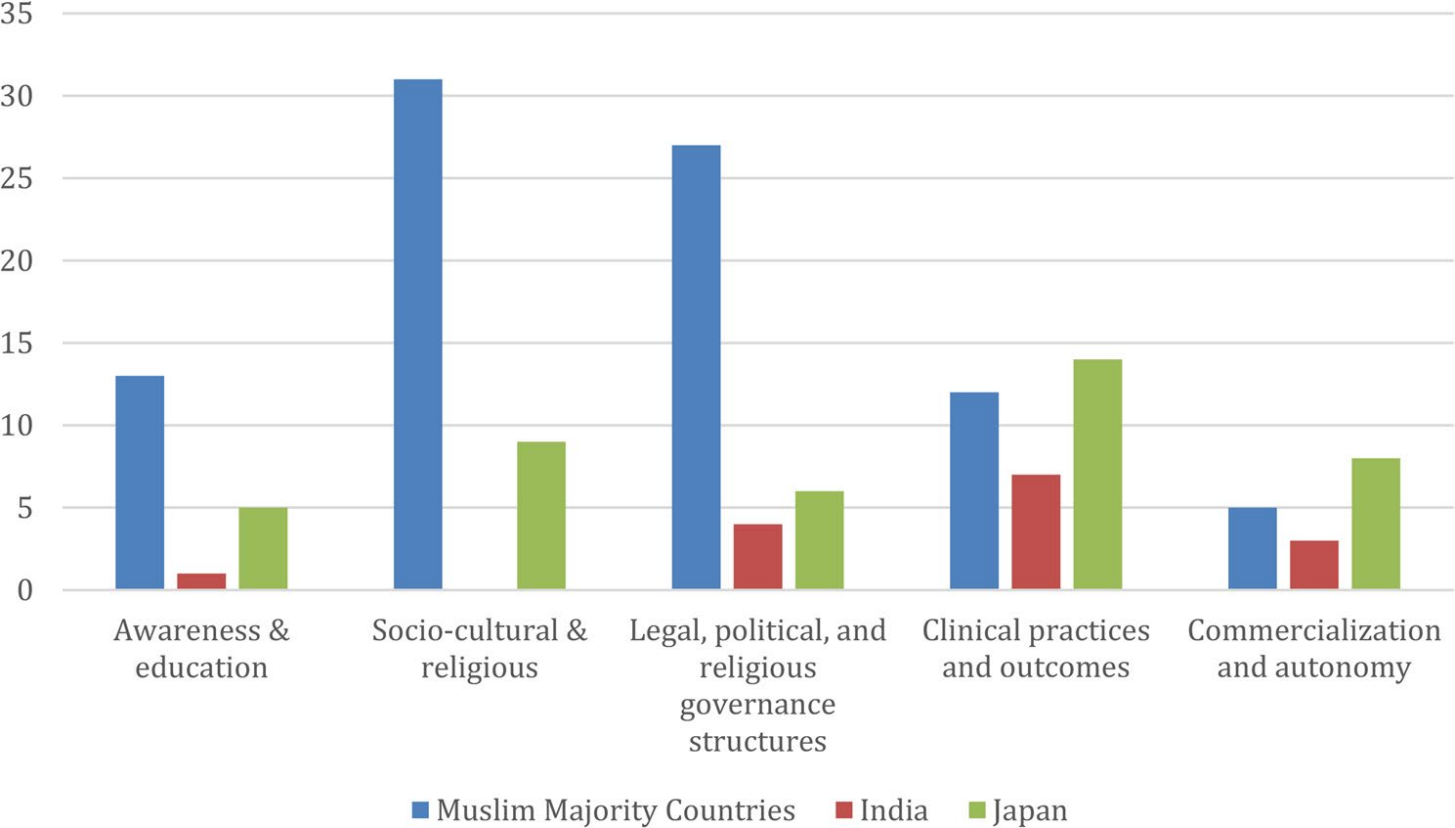
Theme Frequency by Religious Backgrounds. The bar chart below visualizes the frequency of thematic codes assigned to 68 SEF literature documents. Some documents contributed multiple codes, resulting in 115 total coded instances

The overall distribution of the themes for the 68 sources is as shown in Table 3: (1) 12.2%; (2) 24.4%; (3) 23.5%; (4) 26.1%; (5) 13.9%. The articles were less focused on women’s awareness and education nor commercialization and autonomy of SEF, theme 1 and 5. The distribution of themes with respect to religious backgrounds is as shown in Fig. 2. Distribution of themes varied by regional and religious context. Muslim-majority countries had articles predominantly addressing themes 2 and 3 while Japan and India had more articles focused on theme 4. Examining empirical research specifically, studies from India and Japan (11 of 16 empirical studies from these two countries) concentrated on theme 4, whereas empirical studies from Muslim-majority countries more frequently addressed theme 1 (8 of 11 empirical studies from these countries). Within the articles, qualitative studies were notable limited–only three qualitative studies on women undergoing SEF were found—three from Turkey and one from Saudi Arabia, highlighting a gap in research on women’s lived experiences and the decision-making processes of SEF [21, 23, 24]. The other qualitative study was on commercialization of SEF [22]. Though Japan had the most number of articles, we were unable to find a single qualitative study on women undergoing SEF.

#### Theme 1: awareness and education

Studies in this category focused predominantly on empirical surveys and educational interventions aimed at assessing and enhancing knowledge and attitudes toward SEF. Surveys across diverse countries, such as Japan, Egypt, Saudi Arabia, Lebanon, and Iran revealed consistently limited awareness and misconceptions about reproductive aging and fertility preservation options.

Awareness and understanding of SEF varied widely. In Japan, SEF was seen as both women’s empowerment and a countermeasure to demographic decline, but concerns were also raised about delayed marriage, intergenerational burdens—such as children caring for elderly parents—and high-risk pregnancies [25]. Additionally, only 11.5% agreed with the idea that marriage could be postponed for career reasons, while 72.6% felt that recipients of donated eggs should be legally married couples, indicating conservative views about fertility treatment outside of legal marriage [26]. Other researchers found that while more than half of Japanese health sciences university students supported SEF, only 19.9–37.9% could answer knowledge questions correctly [27].

Similar results have been shown for the Muslim-majority countries. In Iran only 6.9% correctly mentioned the low chance of pregnancy after egg freezing at 35 years old [28].

In Saudi Arasbia, 88.1% had heard of SEF, yet only 28.2% felt well-informed [23]. Higher education levels were significantly correlated with greater knowledge. Education and fertility awareness programs improved knowledge significantly, as shown in Egypt [29], where post-intervention knowledge rose from 12.3% to 97.2% and positive attitudes from 25.3% to 92.3%. In Lebanon, single and childless women were more likely to consider SEF [30]. Yet, the lack of information as well as ethical concerns over safety remained key barriers. In Saudi Arabia, 83% of the women undergoing SEF did know about the medical complications associated with SEF, and 46% got their information from media or family and friends [23]. These studies underscore the importance of targeted education to support informed decision-making. Further nuance comes from Japan’s first municipally supported SEF program and found that while lack of a partner or work obligations were common motivations, some women cited endometriosis (18%) or a partner’s illness (15%)—highlighting that SEF often responds to constrained circumstances rather than being purely elective [31]. Across cultural contexts, public misperceptions about age-related fertility decline, risks of late pregnancy, and low awareness of actual success rates persist because of the existing social, cultural, and gendered expectations. These expectations include normative beliefs about appropriate ages for childbearing, intense societal pressure on women to prioritize motherhood (elaborated further in the discussions), and gendered assumptions about women’s primary responsibility for fertility management. In patriarchal contexts, such expectations may also include family pressure regarding marriage timing and concerns about preserving virginity, as seen in the literature in the Islamic contexts [15, 29, 32]. These social norms can prompt some women to consider SEF uncritically and, at the same time, impede informed decision-making, as they may feel pressured to pursue SEF to conform to expectations rather than to exercise an autonomous reproductive choice.

#### Theme 2: Sociocultural and religious perspectives

Most normative articles had significant sections of this theme (34/40). The literature illustrated how societal contexts significantly influence reproductive autonomy and the availability of reproductive technologies. Islamic perspectives, particularly evident in articles that reference Egypt, Qatar, Saudi Arabia, and other Islamic contexts—even when not solely focused on Egypt—underscored ethical debates surrounding the permissibility of elective fertility preservation for unmarried women to preserve their virginity [15–17, 32–36]. These discussions frequently emphasized the preservation of lineage (*nasab*), religious boundaries around marital reproduction, and prohibitions on third-party gametes. While some *fatwas* and scholarly rulings allow SEF for single women under constrained conditions, real-world accessibility remains rare and uneven across jurisdictions [16, 33, 34].

In Saudi Arabia, although 77% of 100 women undergoing SEF did so for social reasons, moral uncertainty—particularly among single women—persisted [23]. In Egypt, two studies referencing the country from outside contexts (Singapore and broader Islamic reviews) highlighted that SEF was often viewed as permissible under Islamic ethics when used within marriage, but SEF for single women remained debated and rarely practiced [16, 17]. Mohamed (2023) documented a dramatic shift in attitudes toward SEF following educational interventions, though the pre-intervention hesitation reflected longstanding religious and social ambivalence [29]. In Lebanon, single, childless women were more open to SEF, though barriers related to stigma, cost, and unclear regulatory guidance persisted [30]. In Qatar, literature continued to emphasize marriage as the legitimate context for childbearing, even as demographic trends strained this expectation [16]. Similarly, qualitative studies from Turkey explored how SEF is portrayed as a form of empowerment yet shaped by conservative gender norms and moral ambivalence about delaying motherhood as transfer of eggs is only permissible after marriage [8, 24]. Göçmen and Kılıç’s (2018) qualitative study with 21 women in Turkey revealed how structural and legal constraints shape experiences of SEF. Although egg freezing is legal for single women, the restriction of embryo transfer to married women created significant emotional stress and uncertainty about future reproductive options, illustrating how legal frameworks can simultaneously enable and constrain reproductive autonomy [24]. Women often pursued SEF not solely as an empowered choice but as a pragmatic response to structural constraints.

While Japan is not governed by religious norms in the same way, it exhibited similar patterns of cultural influence. Nakatsuka (2017) and Kugu (2020) reported that although there is public support for SEF as empowerment and a countermeasure to the birthrate crisis, concerns about delayed marriage and intergenerational burdens—such as children caring for elderly parents—were also prevalent [12, 37]. Beyond the health risks emphasized in Western discourse on late motherhood, these Japanese concerns focus particularly on children being burdened with caring for elderly parents at a young age due to the cultural expectation of filial caregiving (oyakōkō), reflecting Japan’s specific familial obligation structures and rapidly aging population. These findings demonstrate that across secular and religious contexts alike, SEF decisions are deeply embedded in societal expectations and value systems. Sources from India do not suggest any cultural or religious influences on women’s decisions to pursue SEF but concerns for the urban-rural divide and financial barriers were mentioned briefly [38].

#### Theme 3: Legal, political and religious governance

Articles in this theme examined how national laws, religious rulings, institutional guidelines, and government policies shape access to SEF across diverse jurisdictions. These regulatory structures influence not only eligibility criteria—such as age, marital status, or medical indications—but also downstream access to IVF, sperm or egg donation, and legal recognition of stored gametes. The policies of the list of countries are as shown in Table 1.

In India, SEF is legally permissible under the Assisted Reproductive Technology (Regulation) Act 2021 [39]. Interestingly, the legislation even allows unmarried women aged above 21 years to freeze and store their unfertilized eggs(oocytes). However, access to IVF and embryo creation is restricted to married women, creating a significant gap between storage permission and practical utilization [40]. This distinction between oocyte cryopreservation and embryo freezing is ethically and legally significant: egg freezing does not require sperm and can be performed while single, whereas embryo creation typically requires marriage in this regulatory context. However, this could be potentially subject to scrutiny if it is found that the unmarried woman seeking to fertilise her oocytes is engaged in a “live-in” relationship or a *de facto* marriage. Such couples are excluded from accessing services provided under the legislation [41]. As a result, while storage is permitted, actual use is generally limited to married heterosexual couples—reflecting the state’s endorsement of reproduction within a traditional family structure [42]. 

In Japan, there is no law regulating SEF directly, but local governments such as Chiba and Tokyo have introduced subsidy programs that partially cover the cost of freezing [13]. Nonetheless, national-level regulation remains limited, and the Japanese Society of Obstetrics and Gynecology (JSOG) continues to express ethical caution—warning that widespread SEF could delay childbearing and worsen demographic decline [37]. Additionally, IVF and sperm donation remain legally limited to married heterosexual couples, restricting the reproductive options for single women even after oocyte preservation.

In Turkey, SEF is legally available to single women. However, as IVF is only permitted within legal marriage, the downstream use of cryopreserved oocytes is constrained [24]. This discrepancy highlights the tension between allowing women to preserve fertility and limiting their ability to use those eggs later without a husband.

In various Muslim-majority countries, the legal and policy frameworks around SEF are heavily shaped by religious rulings. Fatwas from Saudi Arabia and pan-Islamic institutions (e.g., the International Islamic Fiqh Academy) permit SEF for medical reasons in married women but are largely silent or restrictive regarding single women and non-medical indications [43, 44]. In Egyp**t**, although Dar al-Ifta declared SEF permissible in 2019 for women of childbearing age—regardless of marital status—this permissibility has not been clearly operationalized into policy or practice [35]. Shestak et al. (2023) noted that most Muslim-majority countries still tie fertilization strictly to marriage to preserve lineage (*nasab*) [17]. In Qatar, SEF is not not governed by specific legislation, nor is it explicitly prohibited [16]. In practice, the use of gametes for in vitro fertilization (IVF) is restricted to married couples. While SEF is theoretically available through public health institutions, access is not unconditional; as explained above, certain criteria must be met before women are permitted to undergo the procedure.

Across bottom-quintile GGI countries, SEF is often technically legal but practically constrained. Where access expands, it tends to align with state demographic goals; where restricted, it reflects norms around marriage, gender, or religion. These policies shape not only who can freeze eggs, but also who may use them—highlighting tensions between autonomy, law, and moral governance.

We found no literature addressing the legal limits on storage duration or age criteria for permitting egg freezing. In Japan, Masuya observes that even women over 50 years of age were reluctant to discard frozen eggs, reflecting both enduring hope of motherhood and the lack of alternatives under laws restricting IVF and sperm donation to married couples [45].

#### Theme 4: Clinical practices and outcome

This theme pertains to the practical benefit and the low utility of the specific outcomes of SEF. The largest volume of literature (30/68) focused on clinical outcomes related to SEF, including oocyte retrieval, survival, fertilization, embryo development, and live births. Studies emphasized that effectiveness and safety are influenced by technical factors such as Anti-Müllerian Hormone (AMH) levels, age at freezing, number of oocytes retrieved, and thaw-to-use conversion rates.

Clinical utility of frozen eggs is low in Japan [46–48]. Japan’s largest retrospective study, conducted at a single center, followed 403 women who underwent non-medical oocyte cryopreservation over eight years [46].While most reported satisfaction, only 15% returned to use their eggs, resulting in 13 live births. Another five-year study showed just 11.2% of frozen eggs were used, with a 30.3% live birth rate [48]. Most patients were around age 40, leading to reduced success rates. A municipally subsidized SEF program also found that despite improved access, utilization remained low and follow-up data were limited [31]. These findings underscore that SEF use in Japan is shaped not only by clinical protocols but also by restrictions on IVF and sperm donation for unmarried women [49]. Japan’s largest retrospective study, conducted at a single center, followed 403 women who underwent non-medical oocyte cryopreservation over eight years. While most reported satisfaction, only 15% returned to use their eggs, resulting in 13 live births. Another five-year study showed just 11.2% of frozen eggs were used, with a 30.3% live birth rate. Most patients were around age 40, leading to reduced success rates. A municipally subsidized SEF program also found that despite improved access, utilization remained low and follow-up data were limited.

In Saudi Arabia, Alzahrani et al. (2025) reported that among 100 women who had frozen their eggs for social reasons, only 23% reported concrete plans or intentions to use them in the near future, suggesting uncertainty about future utilization or changing life circumstances [23]. Yet, in India, lower AMH levels, indicating lower ovarian reserve is cited as incentives to SEF [40, 50, 51]. Societal expectations, limited reproductive options for single women, high costs, and procedural burdens all contributed to low utilization. These findings point to the need for better counseling and greater public awareness of SEF’s realistic outcomes.

#### Theme 5: Commercialization and, autonomy

This theme examines how SEF is framed and practiced across different sociopolitical contexts. In countries like Japan, Turkey, India, and Saudi Arabia, SEF is promoted as empowering women to manage fertility. Yet closer scrutiny reveals tensions between autonomy, market forces, and demographic agendas.

In Japan, government subsidies—especially Tokyo’s 2023 program—dramatically increased the number of clinics performing SEF [13, 52]. However, motivations were not always autonomous; many women cited partner illness or parental encouragement as reasons for freezing oocytes, and surveys showed subsidies—not long-term fertility planning—drove uptake [31, 53, 54]. Ethical concerns arise when very young women [18–20] pursue SEF under social or familial pressure [31, 53]. Despite its portrayal of empowerment, SEF often functions as a demographic tool, reinforcing traditional labor norms. SEF serves to retain women in the workforce by enabling them to defer childbearing in the same manner that men can focus on careers without immediate fertility concerns—what Terasawa characterizes as functioning ‘as careless men’—rather than genuinely expanding reproductive freedom or challenging workplace structures that disadvantage caregiving [55]. This critique suggests that SEF may reinforce rather than challenge gender inequalities in workplace expectations and the apparent tension requires clarification.

This framing may seem paradoxical in a context of high gender inequality, where women might be expected to face barriers to workforce participation rather than pressure to remain employed. Japan, however, presents a distinct pattern where women have high labor force participation (over 70%), but tend to work as non-regular workers rather than regular employees, especially after their late thirties, reflecting persistent difficulty in reconciling career advancement with family responsibilities [56]. This structural incompatibility drives declining marriage intentions: approximately 50% of women aged 20–39 cite not wanting to be ‘burdened with work, housework, childcare and caregiving’ as reasons for avoiding marriage—significantly more than men [57]. SEF is thus promoted as enabling women to remain in career-track positions longer by deferring both marriage and childbearing, without addressing the workplace inflexibility and unequal domestic labor that create this dilemma. This represents a distinct manifestation of gender inequality from contexts where women’s workforce participation itself is restricted.

In Turkey, clinic websites framed SEF as an empowered, rational choice by invoking fears of infertility and regret [22]. Yet qualitative studies revealed that women often internalized social anxieties about age and marriage, with access to IVF still restricted to married couples. SEF was marketed as promoting autonomy, but constrained by legal and social barriers [21].

In India, where assisted reproductive technology is more liberally regulated —as evidenced by the ART Act (2021) permitting SEF for unmarried women and allowing commercial surrogacy under certain conditions— SEF intersects with rapid medical tourism and significant economic disparity [42].^3^ How economic disparities shape both the marketing of SEF to urban, affluent women and the limited access for rural and lower-income women requires further empirical investigation to understand whether liberal regulation translates into equitable reproductive autonomy. Critics argue that vague legal provisions risk reinforcing social norms that equate womanhood with motherhood, perpetuating reproductive pressure on women [40]. Moreover, they contend that reproductive autonomy should include the right to oocyte donation without severe restrictions.

Across these countries, SEF is marketed as proactive fertility preservation, but little attention is given to future usage limitations—particularly legal requirements for marriage in embryo creation. Thus, policies claiming to support autonomy may conceal the limited utility of frozen eggs and the societal pressures pushing women toward SEF.

By contrast, in Muslim-majority countries such as Saudi Arabia, Qatar, and Egypt, SEF received the least attention in the literature (Fig. 2) and was largely framed through religious and ethical discourses rather than individualized or commercial narratives. Regulatory frameworks—guided by fatwas from institutions like the Islamic Organization for Medical Sciences (IOMS) and the International Islamic Fiqh Academy (IIFA)—typically restrict SEF to married women and primarily for medical indications [16, 34, 58]. For instance, a 2019 Saudi fatwa permitted oocyte cryopreservation for cancer patients but prohibited fertilization outside legal marriage.

Empirical studies on SEF in Egypt and the Gulf countries remain scarce (only five articles found) and none touched on how women made their decision to undergo SEF as it is practically allowed for married women.

## Discussion

The growing uptake of SEF in countries with bottom-quintile GGI rankings reveals layered ethical tensions—particularly around autonomy, commercialization, and state-driven demographic agendas. While SEF is frequently marketed as empowering, this promise is unevenly realized. In many settings, especially where awareness remains low and research limited (as reflected in Themes 1 and 5), “autonomy” functions more as a rhetorical device than a lived reality.

Several patterns observed mirror Western findings—including that single, childless women are more likely to consider SEF, and concerns about delayed marriage and fertility decline are common such as metioned by Baldwin et al. and Hodes-Wertz et al. [59, 60]. However, critical differences emerge in low gender equality contexts: [1] delayed marriage reflects not just individual preference but rigid gender roles and familial pressures; [2] ‘intergenerational burden’ concerns in Japan uniquely include children caring for elderly parents, reflecting distinct cultural obligations; and [3] legal restrictions limiting IVF to married couples create structural barriers to using frozen eggs that differ from Western settings where partnership is a practical but not legal constraint. These similarities in surface motivations mask deeper contextual differences in the structural conditions shaping reproductive choices.

Given these sociocultural differences, the ethical dimensions of SEF are best addressed not through a top-down application of universal ethical principles or moral theories, but through a bottom-up process that starts with collecting and understanding people’s moral perspectives and preferences. This can be achieved through empirical methods like Patient and Public Involvement and Engagement (PPIE) research, which aims to capture people’s lived experiences and actual moral preferences regarding SEF [61]. The collected data ought then to be analyzed through pluralistic ethical frameworks like Collective Reflective Equilibrium in Practice (CREP) [62, 63]. CREP starts by collecting both public and expert moral preferences and intuitions, screens the preferences/intuitions to remove morally unacceptable biases and social prejudice (e.g. racism), and looks for convergence between public and expert preferences and intuitions, giving weight to those most preferred [62]. The resulting (or laundered) preferences and intuitions are then assessed against relevant normative theories, moral principles and ethical guidelines or declarations with a view to seeking moral consensus between preferences/intuitions on one hand, and ethical theories and principles on the other. Subsequent analysis should reveal which preferences and intuitions are most supported and justified by the normative theories and principles.

Applying PPIE and CREP in SEF contexts would entail collecting public preferences in Muslim-majority societies within different regions and countries (i.e., the Middle East, India) as well as expert medial intuitions regarding SEF. Once collected, the preferences/intuitions would then be screened to remove social bias and prejudice and then assessed against relevant normative theories or principles pertaining to personal autonomy. Ethical analysis should reveal a set of morally justified preferences and intuitions which can then be used for developing public policy for SEF within each country respectively.

Future research into SEF in low gender equality settings must take an empirical approach simply because doing so enables examining not only whether women have formal access to the technology, but how sociocultural expectations of motherhood, family obligations, religious values, and economic constraints actually shape and influence how women experience the prospect of motherhood and make decisions about their reproductive futures.

### Autonomy under pressure: market forces and state agendas

In countries like Japan, Turkey, and India, SEF is promoted as a rational and proactive solution to reproductive aging. Yet, its real-world utility is limited by marriage requirements for embryo use and constrained access to sperm donation. Clinics capitalize on biological clock anxieties, presenting SEF as a form of empowerment while minimizing medical risks and success limitations. This creates a false sense of autonomy where empowerment narratives obscure deeper constraints.

In Japan, government subsidies for SEF (e.g., in Tokyo and Chiba) contributed to a spike in clinics offering the procedure. However, surveys show women were often motivated more by subsidies than by long-term reproductive planning [54]. Many underwent SEF due to societal pressure, sometimes accompanied by family members. Structural barriers—lack of sperm banks, restrictions on single women’s access to fertility treatment—render cryopreserved oocytes unusable without marriage, a fact often omitted in SEF counselling. The framing of SEF as empowerment subtly shifted responsibility for declining birth rates onto women without addressing systemic barriers like rigid work cultures and insufficient childcare support [64].

Western critiques resonate here: Claudia Bozzaro’s model emphasizes that true empowerment requires freedom from social pressure, while Petersen’s “thin ice” critique warns that SEF can easily become a “false hope” technology if aligned with demographic agendas [6, 7]. In lower-quintile countries, SEF often appears as a facade of choice masking deeper societal pressures. Here, relational autonomy—a feminist framework recognizing that autonomous decisions occur within webs of relationships and social contexts rather than in isolation [65] —better describes women’s lived experiences, as their choices are shaped by family, societal, and institutional expectations.

### Muslim-majority countries: restricted but structurally protective

Out of the 29 countries in the lowest GGI quintile, 23 are predominantly Islamic. In most of these Muslim-majority countries, discussions about SEF remain extremely limited. While religious and ethical restrictions do curtail individual reproductive autonomy, they also provide structural protection against aggressive commercialization and the burdens of individualized fertility management. Unlike in Japan, Turkey, or India, women in Muslim-majority contexts are not routinely sold the promise of indefinite fertility preservation, which is often disconnected from actual usage realities. While Turkey is a Muslim-majority country, its more secular legal framework and clinical practices align it more closely with Japan and India in terms of commercialization patterns. Clinic advertisements in these countries rarely mention SEF risks, but the dominant framing centers on marriage, family, and religiously sanctioned reproduction—rather than personal ambition or anti-aging narratives.

This divergence highlights two distinct ethical landscapes: one that promotes autonomy without truly delivering it, and another that restricts autonomy but offers protection against commodification. Ethical evaluations of SEF must be grounded in the lived moral experiences and sociocultural values of each context. Rather than applying universal liberal notions of autonomy, ethical reflection should attend to local meanings shaped by family roles, religious commitments, and community expectations. From this perspective, Islamic regulatory models—though restrictive—may more accurately reflect communal moral reasoning and thus provide ethically coherent reproductive policies within their cultural contexts.

Nonetheless, scholars have argued that SEF could be ethically justified under the category of social necessity [16, 34]. Given declining fertility rates, delayed marriage, and postponed childbirth across many Muslim-majority countries, demand for SEF motivated by social factors may increase in the near future [10]. Although SEF for single women remain largely unrealized in practice, societal attitudes may be slowly shifting, even as awareness, education, and policy clarity remain limited. However, significant barriers to both practice and research persist.^4^ This reluctance reveals the profound gap between theological permission and practical accessibility, shaped by cultural sensitivities and professional concerns about social stigma. As Ghaly (2020) notes, Islamic ethical discussions on SEF are sparse and typically limited to clinical advertisements [16]. Even for these countries, like Japan and Turkey, these advertisements rarely disclose the risks [66, 67] or clarify egg usage restrictions [68] Therefore, careful empirical attention to fertility awareness, commercialization patterns, and women’s actual autonomy is needed before policy shifts can be ethically justified.

### SEF subsidy for distributive justice: autonomy or social engineering?

Japan presents a compelling case study where SEF, though promoted as a tool of empowerment, is deeply intertwined with pronatalist policies. JSOG has long expressed concern that SEF encourages indefinite delays in motherhood, thus potentially exacerbating demographic decline [37]. Historical reliance on external pressures for reform (*gai-atsu*) has led Japan to adopt surface-level gender equity policies while leaving deep-rooted systemic barriers intact [69]. Subsidies for SEF expanded access but primarily positioned women as the agents responsible for solving the fertility crisis. For instance, the 1985 Equal Employment Opportunity Law symbolized progress in gender equality but lacked enforcement mechanisms, contributing to Japan’s persistent low ranking in the Global Gender Gap Index [9, 70, 71].

Generational shifts further complicate this landscape: surveys show that while a growing number of young people (55%) express no desire for children, many still support SEF [72, 73]. This paradox illustrates the influence of *seken*—the societal gaze enforcing conformity to traditional roles [74, 75]. Women often undergo SEF not purely out of autonomous desire but to appear to fulfil societal expectations [76].

Crucially, framing SEF as a technological fix masks its physical and psychological toll. Egg retrieval requires invasive procedures and hormonal interventions, risks that are often minimized in public messaging. Structural inequities intensify these burdens: Japan lacks accessible sperm banks, and fertility treatments for single women remain largely prohibited. Yet the onus of falling fertility is placed squarely on women, as highlighted by a recent proposal from a conservative lawmaker suggesting mandatory hysterectomy for women over 30 to encourage earlier childbirth [77]. Such rhetoric underscores the disproportionate reproductive burden imposed on women.

Moreover, the legal restriction of ART to married couples renders many cryopreserved oocytes unusable unless women eventually marry—a limitation rarely disclosed in SEF marketing. As such, SEF in Japan risks becoming a kind of temporary cosmetic solution: one that shields women from social scrutiny without meaningfully expanding reproductive autonomy. Feminists in Japan, while acknowledging the empowerment, has criticized this as selling image of liberty in exchange of self-harm (both physical and financial) as a “trap” [78].

### Women’s autonomy and ethical considerations in lower-quintile countries

In Muslim-majority contexts reproductive technologies are primarily sanctioned to facilitate childbearing within marriage, with no recognition of single-parent reproduction. Although Islamic ethics, such as those expressed in the *International Islamic Code for Medical and Health Ethics*, emphasize that autonomy is not differentiated by sex, the notion of reproductive autonomy—especially as conceptualized in Western secular discourse—finds little support either in Islamic normative thought, in prevailing social practices, or in codified laws adopted by Muslim-majority countries. Married couples themselves are not unrestricted in their choices, as the use of third-party reproduction (e.g., sperm donation, egg donation, surrogacy) remains prohibited [8, 21, 26]. These normative boundaries challenge the idea of SEF as an unqualified reproductive right. Instead, elective SEF is often regarded with suspicion, given concerns about disrupting kinship and religiously sanctioned family formation. This model prioritizes communal coherence over individual preference and stands in contrast to commercial narratives elsewhere that frame SEF as a tool for personal empowerment.

Thus, while some countries appear to offer women reproductive freedom through access to SEF, the autonomy granted may be more illusory than real. Women themselves were concerned that SEF might delay marriage, which conflicts with societal priorities for early marriage—though changing—recent survey from Jorden showed that women believed that SEF would alleviate the psychological burden of late marriage and fear of future partner rejection [79].^5^ Therefore, SEF is now a tool for increasing marriagibility. Having SEF as an “option” does not necessarily equate to genuine empowerment, particularly when its use is accompanied by social or professional pressures that push women who wish to become mothers, is tightly regulated, or if the broader social and religious frameworks fundamentally constrain its application. This reiterates the point that SEF risks becoming a false sense of autonomy—a symbolic gesture towards empowerment without its practical realization.

This tension is particularly evident when SEF is introduced alongside aggressive commercialization and Western-style marketing strategies. Fertility clinics in many lower-quartile countries promote SEF using images of Western role models and empowerment narratives, often minimizing or omitting the medical risks and future usage limitations. In some Middle Eastern and North African (MENA) countries, information about risks is virtually absent, while in parts of Asia, benefits are emphasized disproportionately. This marketing imbalance reflects a deeper conflict: commercial incentives often override patient education.

Government policies can also obscure ethical concerns. Subsidies for young women—sometimes as young as 18—are framed as expanding reproductive choice, yet such decisions may be made without full understanding of SEF’s limitations [13]. Meanwhile, in Muslim-majority countries, SEF may be legally permitted but functionally restricted to married women, reinforcing communal norms.

These findings highlight the urgent need for more culturally sensitive, context-specific discussions about SEF, moving beyond Western-centric notions of autonomy. Global bioethics must engage more seriously with relational autonomy frameworks that acknowledge how reproductive decisions are made not only by individuals but also within family, community, and religious structures.

### Limitations of the study

This study has several limitations. First, language and database coverage may have excluded relevant publications from bottom quintile GGI countries, particularly in Sub-Saharan Africa. While English, Japanese, and Arabic databases were systematically searched, French-language databases were not included, potentially missing literature from Francophone African countries such as Benin, Burkina Faso, Côte d’Ivoire, Niger, Mali, Guinea, Chad, and the Democratic Republic of Congo. Similarly, publications in Portuguese, Swahili, and Urdu were not accessible, limiting our understanding of SEF discourse in countries such as Mozambique, Pakistan, and other African nations. Future reviews should incorporate multilingual search strategies to ensure more comprehensive geographic coverage [80].

Second, the scarcity of empirical data—particularly qualitative research—on SEF in bottom-quintile GGI countries constrained the depth of analysis around women’s lived experiences and decision-making processes. Much of the material came from normative reviews, policy documents, and grey literature, which, while contextually valuable, lack the rigor of structured interviews or large-scale surveys. Only four qualitative studies exploring women’s experiences with SEF were identified globally, three from Turkey and one from Saudi Arabia, representing a significant gap in understanding how women navigate SEF decisions across diverse cultural contexts [81].

Third, due to limited empirical research in many Middle Eastern, North African (MENA), and South Asian countries, the study drew on documents from nearby regions to provide context. While this broadened regional understanding, it may have introduced assumptions not fully reflective of each country’s specific sociocultural dynamics. For Japan, the overrepresentation of commentaries and conference reports similarly limited deeper analysis of SEF’s individual and societal implications. With only four qualitative studies total—three from Turkey and one from Saudi Arabia—and predominantly normative rather than empirical sources from Muslim-majority countries, we could characterize the regulatory and religious frameworks but could not provide the same depth of analysis regarding women’s lived experiences. Notably, while Japan’s extensive literature (23 publications) focused on quantitative outcomes reflecting pronatalist policy interests, the qualitative studies from Turkey and Saudi Arabia centered on understanding women’s actual experiences and decision-making. This disparity highlights the urgent need for qualitative empirical research to deepen understanding of women’s experiences undergoing SEF across diverse contexts [82].

Fourth, we did not systematically track whether women who freeze eggs eventually discard them, a question with significant ethical implications regarding resource allocation, psychological impacts, and the practical utility of SEF. Future research should examine the full lifecycle of frozen oocytes, including disposal decisions and their emotional consequences.

Lastly, the focus on countries in the lowest quintile of the Gender Gap Index may limit generalizability to other contexts. However, this focus was intentional to examine SEF in settings where reproductive autonomy is most constrained, providing insights into technology intersects with structural gender inequality. Future research should expand geographical scope and adopt more robust qualitative and quantitative approaches to better capture SEF’s complex ethical and policy dimensions across diverse contexts.

## Conclusion

SEF policies must address cultural, educational, and practical realities rather than merely promoting autonomy as delaying motherhood for career goals. Meaningful autonomy in diverse global contexts demands culturally aware regulations, transparent clinical disclosures, and comprehensive fertility education. Without such frameworks, SEF risks becoming another burdensome demographic intervention rather than genuine reproductive empowerment.

## Supplementary Information

Supplementary Material 1

Supplementary Material 2

Supplementary Material 3

## Data Availability

All data generated or analyzed during this study are included in this published article and its supplementary information files. Additional File 1 lists the 29 bottom-quintile Gender Gap Index countries and their characteristics. Additional File 2 provides the complete reference list of all 68 sources included in the scoping review. Additional File 3 presents the thematic categorization of each source by country, author, source type, and assigned themes. The search strategies and PRISMA flow diagram are included in the main manuscript. No primary datasets were generated as this is a scoping review of existing published literature.

## Abbreviations

SEF: Social Egg Freezing
GGI: Gender Gap Index
ART: Assisted Reproductive Technology
CREP: Collective Reflective Equilibrium in Practice
IVF: In Vitro Fertilization
JSOG: Japan Society of Obstetrics and Gynecology
MENA: Middle East and North Africa
JBI: Joanna Briggs Institute
IIFA: International Islamic Fiqh Academy
AMH: Anti-Müllerian Hormone

## Footnotes

1 Feminist Association of Bioethics Congress, was held in Doha, Qatar on June 2, 2024.

2 Two sources addressed Islamic bioethical perspectives across multiple Muslim-majority countries rather than focusing on a single nation, and are categorized as ‘Multi-country Islamic perspectives’ in our analysis.

3 Medical tourism refers to individuals traveling to other countries to access medical procedures, often at lower costs. India has become a major destination for reproductive tourism due to its advanced fertility services, lower costs compared to Western countries, and relatively permissive regulatory environment.

4 During our data collection, physicians working in fertility clinics in Qatar declined to disclose even their clinic names or confirm whether they offered SEF, despite theoretical religious permissibility.

5 This survey was not included in our review as it had been published after our submission.
