# Supplementary material for: Autonomy under pressure: a scoping review of social egg freezing in the bottom quintile of the gender gap index
Source: BMC Med Ethics. 2026 Jan 5;27:47. doi: 10.1186/s12910-025-01353-8 (PMC12955265; doi:10.1186/s12910-025-01353-8)
Supplement: Supplementary file 1 — Supplementary Material 1 [file 12910_2025_1353_MOESM1_ESM.docx]

| **#** | **Country** | **Author(s) & Year** | **Source Type** | **Theme(s)** |
| --- | --- | --- | --- | --- |
| 1 | Algeria | Julud S, 2022 | Peer-reviewed, Normative | 3 |
| 2 | Egypt | Chin AHB SSM. 2023 | Peer-reviewed, Normative | 2,3 |
| 3 | Egypt | Chin AHB MS . 2022 | Peer-reviewed, Normative | 2,3 |
| 4 | Egypt | Mohamed N 2023 | Peer-reviewed, Empirical | 1 |
| 5 | Egypt | Hasab Allah MF. 2021 | Peer-reviewed, Normative | 1 |
| 6 | Egypt | Egypt's Dar Al-fta | Gray literature, Normative | 2 |
| 7 | Egypt | Abdul Maqsood MGMM.2024 | Peer-reviewed, Normative | 2,3 |
| 8 | Egypt | Al-Kareem AGAG. 2019 | Peer-reviewed, Normative | 3,4 |
| 9 | Egypt | Abdel Hafeez AKA.2024 | Peer-reviewed, Normative | 2,3 |
| 10 | Egypt | Al-Koomi AMRAS. 2022 | Peer-reviewed, Normative | 2,3 |
| 11 | Egypt | Al-Jarhi MMM.2019 | Peer-reviewed, Normative | 2,4 |
| 12 | Egypt | Taya HHIH. 2023 | Peer-reviewed, Normative | 2 |
| 13 | Egypt | Abdullatif RAF. 2022 | Peer-reviewed, Normative | 2 |
| 14 | Egypt | Al-Masir.2019 | Peer-reviewed, Normative | 2,3 |
| 15 | Egypt | Al-Barbari BSA. 2024 | Peer-reviewed, Normative | 3 |
| 16 | India | Mahajan NK. 2025 | Peer-reviewed, Normative | 4 |
| 17 | India | Allahbadia GN. 2016 | Peer-reviewed, Normative | 4,5 |
| 18 | India | Bärnreuther S. 2021 | Peer-reviewed, Normative | 3,4,5 |
| 19 | India | Jindal UN. 2018 | Peer-reviewed, Normative | 4 |
| 20 | India | Chatwal J 2023 | Peer-reviewed, Empirical | 4 |
| 21 | India | Desai S. 2025 | Peer-reviewed, Normative, Review | 3,4, |
| 22 | India | Rana R. 2021 | Peer-reviewed, Normative, Review | 3,5 |
| 23 | India | Tyagi P.2025 | Peer-reviewed, Empirical | 4 |
| 24 | India | Mishra R. 2023 | Peer-reviewed, Normative | 3 |
| 25 | India | *The Assisted Reproductive Technology (regulation) Act* *2021* | Gray literature, Normative | 3 |
| 26 | Iran | Hafezi M. 2022 | Peer-Review, Empirical | 1,4 |
| 27 | Iran | Akhnodi MM 2023 | Peer-Review, Empirical | 1 |
| 28 | Islamic Countries | Shestak VA, 2023 | Non-Peer Review, Normative | 2,3 |
| 29 | Islamic Countries | Al-Bar MA 2015 | Non-Peer Review, Normative | 2,3 |
| 30 | Japan | Asai Y. et al. 2020 | Peer-Review, Empirical | 4 |
| 31 | Japan | Matsuya A, 2014 | Non-Peer Review, Normative | 3,4,5 |
| 32 | Japan | Kikuchi I, 2018 | Peer-reviewed, Empirical | 1,2,4 |
| 33 | Japan | Kikuchi I, 2018 | Peer-reviewed, Empirical | 4 |
| 34 | Japan | Kawakami A. 2020 | Peer-reviewed, Empirical | 1 |
| 35 | Japan | Hibino Y. 2014 | Non-peer reviewed, Normative | 2 |
| 36 | Japan | Umahayashi K. 2013 | Peer-reviewed, Normative | 1 |
| 37 | Japan | Terasawa S. 2024 | Peer-reviewed, Normative | 2,5 |
| 38 | Japan | Kugu K. 2020 | Non-peer reviewed, Normative | 2,4,5 |
| 39 | Japan | Nakatsuka M. 2014 | Peer-reviewed, Empirical | 2,3,4 |
| 40 | Japan | Nakatsuka M.2015 | Peer-reviewed, Empirical | 5 |
| 41 | Japan | Nakatsuka M. 2017 | Peer-reviewed, Empirical | 1 |
| 42 | Japan | Okuyama N. 2022 | Peer-reviewed, Empirical | 4 |
| 43 | Japan | Takahashi T. 2022 | Peer-reviewed, Normative | 2,4 |
| 44 | Japan | Shirasawa H. et al. 2023 | Peer-reviewed, Empirical | 5 |
| 45 | Japan | Yoshinaga K. et al. 2023 | Peer-reviewed, Empirical | 4 |
| 46 | Japan | Ohno M. et al 2024 | Peer-reviewed, Empirical | 2,3,4 |
| 47 | Japan | Tokyo Metropolitan Government 2023 | Gray-literature, Normative | 3 |
| 48 | Japan | Tokyo Metropolitan Government Welfare Bureau 2023 | Gray-literature, Empirical | 2,3,4,5 |
| 49 | Japan | Tokyo Metropolitan Government Welfare Bureau, Child and Family Support 2023 | Gray-literature, Empirical | 2,3,4,5 |
| 50 | Japan | Tokyo Metropolitan Government Welfare Bureau 2024 | Gray-literature, Empirical | 4,5 |
| 51 | Japan | Japanese Society of Obstetrics and Gynecology(JSOG). 2021 | Gray-literature, Empirical | 1,4 |
| 52 | Jordan | Al-Bāz AA. 2014 | Peer-reviewed, Normative | 2,3 |
| 53 | Lebanon | Ghazeeri G. et al. 2023 | Peer-reviewed, Empirical | 1 |
| 54 | Qatar | Ghaly M, 2020 | Non-peer reviewed, Normative | 3,2 |
| 55 | Qatar | Qatar Foundation 2025 | Gray-literature, Normative | 4 |
| 56 | Saudi Arabia | Shibata M. 2022 | Non-peer reviewed, Normative | 5 |
| 57 | Saudi Arabia | Saadia Z, et al. 2024 | Peer-reviewed, Empirical | 1 |
| 58 | Saudi Arabia | Alzahrani FA, et al. 2025 | Peer-reviewed, Empirical (Qualitative +Quantitative) | 1,2,4 |
| 59 | Saudi Arabia | Muaygil R, 2023 | Peer-reviewed, Normative | 3,4,5 |
| 60 | Saudi Arabia | Policy of the Fatwa 2019 | Gray-literature: Islamic Policy | 2,3 |
| 61 | Saudi Arabia | Al-Ghamdi BAA 2024 | Gray-literature, Normative | 2,3 |
| 62 | Turkey | Dundar Akın O, Boza A, Yakin K, Urman B. 2019 | Peer-reviewed, Empirical | 1 |
| 63 | Turkey | Seyhan A, Ata B, Uncu G, Yildiz S, Gidener T. 2021 | Peer-reviewed, Empirical | 1,4 |
| 64 | Turkey | Cil AP, Seli E. 2013 | Peer-reviewed, Normative Review | 4 |
| 65 | Turkey | Kılıç A. 2024 | Peer-reviewed, Empirical (Qualitative) | 5 |
| 66 | Turkey | Göçmen İ, Kılıç A. 2018 | Peer-reviewed, Empirical | 2,5 |
| 67 | Turkey | Köroğlu N, Aydın T. 2023 | Peer-reviewed, Normative Review | 4 |
| 68 | Turkey | Kılıç A, Göçmen İ. 2018 | Peer-reviewed, Empirical (Qualitative) | 2,5 |

Additional File 3. Overview of records reviewed by country, author, source type and respective theme(s)
