# Supplementary material for: Autonomy under pressure: a scoping review of social egg freezing in the bottom quintile of the gender gap index
Source: BMC Med Ethics. 2026 Jan 5;27:47. doi: 10.1186/s12910-025-01353-8 (PMC12955265; doi:10.1186/s12910-025-01353-8)
Supplement: Supplementary file 2 — Supplementary Material 2 [file 12910_2025_1353_MOESM2_ESM.docx]

# **Additional File 1. Lower Quintile of Gender Gap Index Countries 2024.**

# **Their ART, SEF Policies, and Cultural Contexts** (4)

| **Rank**  **(#articles)** | **Country** | **Predominant Religion** | **SEF Policy** | **Approximate SEF Cost (USD)** | **ART Law and Cultural Attitude** |
| --- | --- | --- | --- | --- | --- |
| 118  (23) | Japan | Shintoism/Buddhism (Secular) | Allowed; local government subsidies for egg freezing in some areas (up to $2600)(52) | $2,000–$3,000 per cycle (plus storage) | Guidelines-based; SEF and ART allowed, culturally cautious toward non-traditional families |
| 119  (0) | Comoros | Islam | No known policy; ART access extremely limited | Unknown / Not available | No ART law; conservative Islamic context restricts ART |
| 120  (0) | Burkina Faso | Islam | No policy; limited ART infrastructure | Unknown / Not available | No clear ART law; access minimal, religious conservatism affects attitudes |
| 121  (0) | Côte d'Ivoire | Islam/Christianity | No policy; limited ART access | Unknown / Not available | No ART regulation; mixed religious influence, low ART awareness |
| 122  (0) | Sri Lanka | Buddhism | Permitted; no specific SEF regulation | $4400–$5100 per cycle (53) | Permitted under regulation; Buddhist-majority, moderate ART acceptance |
| 123  (1) | Jordan | Islam | Permitted under ethical and religious guidelines; limited SEF discussion | $2,000–$4,000 per cycle | Permitted with restrictions; Islamic bioethics influence ART use |
| 124  (0) | Bhutan | Buddhism | No policy; ART not widely practiced | Unknown / Not available | No ART law; limited services, traditional Buddhist family values |
| 125  (0) | Nigeria | Islam/Christianity | Permitted; SEF discussed in private clinics, no regulation | $1,500–$2,500 (private sector) | Permitted; private clinics lead, cultural diversity affects ART acceptance |
| 126  (5) | Saudi Arabia | Islam | Permitted for medical reasons; elective SEF restricted | $3,000–$5,000 per cycle | Strictly regulated; ART for married couples only under Islamic law |
| 127  (7) | Türkiye | Islam | Permitted for medical and SEF strictly regulated (diminished ovarian reserve, women above 38, and family history of premature menopause)(54) | $4,400 to $5,500 USD (55) | Permitted under Ministry of Health guidance; SEF allowed for anticipated infertility; cultural tension remains |
| 128  (0) | Fiji | Christianity | No clear policy; ART limited | Unknown / Not available | Minimal regulation; Christian-majority, traditional family norms dominate |
| 129  (10) | India | Hinduism | Permitted; regulated under ART Act (2021), SEF allowed for unmarried women | $1,000–$2,500 per cycle (56) | ART Act (2021) governs use; socially stratified access, SEF legal |
| 130  (2) | Qatar | Islam | Restricted; ART available to married couples, SEF uncommon | $3,000–$6,000 per cycle | Highly restricted; ART allowed for married couples only |
| 131  (0) | Kuwait | Islam | No clear SEF policy; ART for married couples only | $3,000–$5,000 per cycle | Restricted to married couples; cultural resistance to SEF |
| 132  (0) | Maldives | Islam | No policy; ART limited and culturally sensitive | Unknown / Limited access | No ART law; Islamic norms limit ART discussion |
| 133  (1) | Lebanon | Islam/Christianity | Permitted; SEF discussed, private clinic access | $4,000-5000 maximum per cycle(16) | Permitted; religiously diverse, private sector led, growing acceptance |
| 134  (0) | Benin | Christianity | No policy; ART infrastructure minimal | Unknown / Not available | No formal regulation; minimal access, traditional norms |
| 135  (14) | Egypt | Islam | Permitted for medical reasons; social egg freezing not widely practiced | $1,500–$3,000 per cycle (private clinics) | Permitted under Islamic guidelines; social stigma limits openness |
| 136  (0) | Oman | Islam | ART restricted to married couples; SEF culturally sensitive | $3,000–$5,000 per cycle | Restricted to married couples; ART under Islamic values |
| 137  (0) | Morocco | Islam | Permitted for married couples; SEF not regulated | $2,000–$4,000 per cycle | Permitted under restrictions; Islamic and traditional norms |
| 138  (0) | Niger | Islam | No policy; ART services limited | Unknown / Not available | No regulation; conservative context limits ART |
| 139  (0) | Algeria | Islam | Permitted under certain restrictions; SEF not regulated | $2,000–$3,500 per cycle | Permitted; Islamic law influences access and use |
| 140  (0) | Democratic Republic of the Congo | Christianity | No policy; ART infrastructure very limited | Unknown / Not available | No ART law; infrastructure and cultural barriers |
| 141  (0) | Mali | Islam | No clear policy; ART services scarce | Unknown / Not available | No policy; conservative, ART rarely discussed |
| 142  (0) | Guinea | Islam | No policy; ART infrastructure minimal | Unknown / Not available | No regulation; very limited access |
| 143  (2) | Iran (Islamic Republic of) | Islam | Permitted under Islamic bioethical framework; SEF limited | $1,200–$2,500 per cycle | Permitted under Islamic law; strong state ART support |
| 144  (0) | Chad | Islam/Christianity | No policy; limited ART services | Unknown / Not available | No ART regulation; conservative cultural setting |
| 145  (0) | Pakistan | Islam | Permitted; SEF emerging in private sector, not regulated | $1,000–$2,000 per cycle (private clinics) | Permitted; Islamic ethics frame acceptance, SEF emerging |
| 146  (0) | Sudan | Islam | No policy; ART highly restricted | Unknown / Not available | No ART law; ART viewed as culturally sensitive |
