# Supplementary material for: Autonomy under pressure: a scoping review of social egg freezing in the bottom quintile of the gender gap index
Source: BMC Med Ethics. 2026 Jan 5;27:47. doi: 10.1186/s12910-025-01353-8 (PMC12955265; doi:10.1186/s12910-025-01353-8)
Supplement: Supplementary file 3 — Supplementary Material 3 [file 12910_2025_1353_MOESM3_ESM.docx]

**Additional File 2: Records used for this review**

**Turkey**

1. **Kılıç A.** Medical markets for imagined futures: the framing of egg freezing on fertility clinic websites in Turkey. *BioSocieties.* 2024;19(3):402–23.
2. **Göçmen İ, Kılıç A.** Egg freezing experiences of women in Turkey: From the social context to the narratives of reproductive ageing and empowerment. *Eur J Womens Stud*. 2018;25(2):168–82. doi:10.1177/1350506817742929.
3. **Köroğlu N, Aydın T.** Oocyte vitrification for oncological and social reasons. *Turk J Obstet Gynecol.* 2023;20(1):59–63. doi:10.4274/tjod.galenos.2022.59827
4. **Kılıç A, Göçmen İ.** Fate, morals and rational calculations: Freezing eggs for non-medical reasons in Turkey. *Soc Sci Med.* 2018;203:19–27. doi:10.1016/j.socscimed.2018.03.014
5. **Dundar Akın O, Boza A, Yakin K, Urman B**. Awareness of fertility and reproductive aging in women seeking oocyte cryopreservation, reproductive aged controls, and female health care professionals: a comparative study. *Eur J Obstet Gynecol Reprod Biol*. 2019;233:146–50. doi:10.1016/j.ejogrb.2018.12.009. PMID: 30597339.
6. **Seyhan A, Ata B, Uncu G, Yildiz S, Gidener T.** A survey of women who cryopreserved oocytes for non-medical indications (social fertility preservation). *Reprod Sci.* 2021;28(8):2216–22. doi:10.1007/s43032-021-00460-2
7. **Cil AP, Seli E.** Current trends and progress in clinical applications of oocyte cryopreservation. *Curr Opin Obstet Gynecol.* 2013;25(3):247–54. doi:10.1097/GCO.0b013e32836091f4

**Saudi Arabia**

1. **Shibata M.** "Sentaku dekiru koto" o ouka suru: Sauji Arabia no josei-tachi [Enjoying the Ability to Choose: Women in Saudi Arabia]. Tokyo: Japan Center for Cooperation with the Middle East (JCCME); 2022 Nov. Available from: [https://www.jccme.or.jp/download. Accessed 2025 Jul 4](https://www.jccme.or.jp/download.%20Accessed%202025%20Jul%204).
2. **Saadia Z, AlHarbi A, Almutairi F, AlOmar A, AlEnezi H, AlRashidi R, et al.** Knowledge, attitudes, and perceptions of women of reproductive age regarding fertility and elective oocyte cryopreservation: A study from the Al-Qassim Region. *Cureus.* 2024;16(9):e69903. doi:10.7759/cureus.69903
3. **Muaygil R.** Motherhood, fairness, and flourishing: Widening reproductive choices in Saudi Arabia. *Camb Q Healthc Ethics.* 2023;32(2):276–88. doi:10.1017/S0963180122000585
4. **Alzahrani FA, Alghamdi AA, Alsulaiman NA, Alarfaj AA, Alotaibi AS, AlKhaldi AS, et al.** Experiences and perspectives of women undergoing oocyte cryopreservation in Riyadh, Saudi Arabia: A mixed-methods study. *Int J Womens Health.* 2025;17:879–95. doi:10.2147/IJWH.S496633
5. **Policy of the fatwa**, Senior Council of Scholars, Resolution no. 245, 2019.
6. **Al-Ghamdi BAA.** Tajmid al-buwaydat ghayr al-malqaha "bi-al-tajfif aw al-tazjij": Ru’yah fiqhiyyah [Freezing unfertilized oocytes by dehydration or vitrification: A jurisprudential perspective]. *Majallat Jami‘at al-Malik Khalid lil-‘Ulum al-Shar‘iyyah wa-al-Dirasat al-Islamiyyah.* 2024;21(4):103–57. Available from: <http://search.mandumah.com/Record/1516738>

**Qatar**

1. **Ghaly M.** Islamic ethics and infertility treatment. Doha, Qatar: world innovation summit for health (WISH). Middle East Fertility Society Journal. 2020.
2. **Qatar Foundation.** Embryologist from QF’s Sidra Medicine explains what egg freezing offers to women [Internet]. Doha: Qatar Foundation; [date unknown]. Available from: [https://www.qf.org.qa/stories/embryologist-from-qfs-sidra-medicine-explains-what-egg-freezing-offers-to. Accessed 2025 Jul 4](https://www.qf.org.qa/stories/embryologist-from-qfs-sidra-medicine-explains-what-egg-freezing-offers-to.%20Accessed%202025%20Jul%204).

**Lebanon**

1. **Ghazeeri G, Saad R, Hachem H, Khoury S, Abbas HA, Harfouche M, et al.** Knowledge and attitudes toward fertility preservation (medical and social freezing) among Lebanese women between the ages of 18 and 39 years. *PLoS One.* 2023;18(9):e0291249. doi:10.1371/journal.pone.0291249

**Jordan**

1. **Al-Bāz AA.** Tajmīd al-ḥayawānāt al-manāwīyah wa-al-buwayḍāt ruʾyah fiqhiyyah ṭibbiyyah [Sperm and ovum cryopreservation: medical and jurisprudential perspective]. *Dirāsāt ʿUlūm al‑Sharīʿah wa‑al‑Qānūn.* 2014;41(1):215–34.

**Japan**

1. **Ohno M, Kikuchi I, Kagawa N, et al.** The importance of social oocyte cryopreservation in supporting local municipalities: a prospective study. *Women’s Health (Lond)*. 2024;20:17455057241276256. doi:10.1177/17455057241276256.
2. **Yoshinaga K, Hashimoto T, Fukuoka Y, Okuyama N, Kyono K.** Non-medical oocyte cryopreservation at a single center in Japan: 8 years of experience. *Reprod Med Biol*. 2023;22(1):e12549. doi:10.1002/rmb2.12549.
3. **Shirasawa H, Kumazawa Y, Sato W, et al.** The first nationwide website survey of the availability and costs of medical and non-medical oocyte cryopreservation in Japan. *Heliyon*. 2023;9(8):e18071. doi:10.1016/j.heliyon.2023.e18071.
4. **Okuyama N, Oka N, Aono N, Tsunoda M, et al.** A study in status of use and live birth rates of cryopreserved oocytes for social reasons. *Hum Reprod.* 2022;37(Suppl 1):deac107.416. doi:10.1093/humrep/deac107.416
5. **Nakatsuka M.** Nationwide survey on attitude toward social egg freezing: social influence on women’s empowerment and increase in the average age of childbirth. In: Vol 0300-9165. ; 2017:600-601.
6. **Nakatsuka M.** Awareness of various issues related to oocyte donation: findings from a nationwide survey of the general public. *Nihon Sanka Fujinka Gakkai Zasshi*. 2015;67(2):746.
7. **Nakatsuka M.** Mikon josei no shakaiteki tekiō ni yoru ranshi tōketsu hozon e no ishiki to jittai: sanfujinka shisetsu daihyōsha e no zenkoku chōsa [Attitudes and realities toward social egg freezing among unmarried women: a national survey of obstetrics and gynecology facility representatives]. In: *2014 Annual Meeting Abstracts of the Japan Society of Obstetrics and Gynecology*; 2014. Abstract No. 128.
8. **Kawakami ,** **Asazawa K.** Knowledge and positive consciousness of oocyte freezing among female and male of reproductive age, mainly health sciences university students. *Kiryu Univ Bull*. 2020;1:129–136.
9. **Kugu K**. Oocyte cryopreservation for fertility preservation: predicted benefit with serious pitfalls. *Acta Obstet Gynaecol Jpn*. 2020;72(10):1157–1164.
10. **Takahashi T.** Exploring infertility biologically and sociologically: the hidden risks and benefits of oocyte freezing. *Fukushima Med J*. 2022;72(1):1–7. doi:10.5387/fmedj.72.1_1.
11. **Terasawa S.** “Funin chiryo to shigoto no ryōritsu” kenkyū no kadai to tenbō: iryō, koyō no ryōiki ni okeru jendā baiasu no kōsa ni chakumoku shite [Challenges and prospects of research on balancing infertility treatment and work: focusing on intersecting gender bias in medical and employment domains]. *Nihon Rōmu Gakkai-shi*. 2024;25(2):56–69. doi:10.24592/jshrm.25.2_56.
12. **Umahayashi K.** “Review on the present state of support agencies for the treatment of sterile women” and “ovum preservation guideline index”- a contents review. *Kiryu Univ Bull*. 2013;24:127–129.
13. **Hibino Y.** Globalization of reproductive technologies and Japan. *Igaku Tetsugaku Igaku Rinri*. 2014;32:90–95.
14. **Asai Y, Nukui R, Terawaki N, Komiya S, Himeno T, Inoue T, Morimoto Y.** Current status and patient characteristics of social fertility preservation (oocyte cryopreservation): a report from an ART clinic. *Nihon Sanka Fujinka Gakkai Zasshi*. 2020;72(Suppl):S-619.
15. **Kikuchi I, Miyakuni Y, Suzuki I, et al.** The reality of the first oocyte cryopreservation project funded by local government: who truly benefits? *Nihon Sanka Fujinka Gakkai Zasshi*. 2018;70(2):732.
16. **Kikuchi I, Kagawa N, Shirosaki Y, et al.** Early outcomes of a municipally funded oocyte cryopreservation programme in Japan. *Hum Fertil (Camb)*. 2018;22(4):266–272. doi:10.1080/14647273.2018.1464215.
17. **Matsuya A.** Ugokidasita mikon josei no ranshi touketsu hozon [A move has begun towards egg freezing for single women]. *Nikkei Med*. 2014 Feb:36–37.
18. **Tokyo Metropolitan Government.** [Start of Financial Assistance for Egg Freezing Costs; in Japanese]. Tokyo Metropolitan Government. September 15, 2023. Accessed March 4, 2025. <https://www.metro.tokyo.lg.jp/tosei/hodohappyo/press/2023/09/15/05.html>
19. **Tokyo Metropolitan Government, Welfare Bureau.** “Ranshi tōketsu e no shien no kentō ni kansuru jōkyō chōsa kekka” [Survey results on the current status of considerations for supporting egg freezing]. Published September 2023. Accessed [October 3, 2024]. Available from: <https://www.fukushi.metro.tokyo.lg.jp/kodomo/shussan/ranshitouketsu/touketsu/joukyouchousa.files/ranshi-chousakekka.pdf>
20. **Tokyo Metropolitan Government, Welfare Bureau, Child and Family Support Division.** “Mirai ni tsunagaru sentakushi: minna de issho ni shiritai ranshi tōketsu no koto” [Future-connected choices: learning together about egg freezing]. Published 2024. Accessed [October 10, 2024]. Available from: <https://www.fukushi.metro.tokyo.lg.jp/documents/d/fukushi/ranshi_tebiki_2>
21. **Tokyo Metropolitan Government, Welfare Bureau.** “Ranshi tōketsu e no shien ni muketa chōsa jigyōtō roku iryō kikan ichiran: Reiwa 6 nen 8 gatsu 20 ka jiten” [List of registered medical institutions for the survey project on support for egg freezing (as of August 20, 2024)]. Published 2024. Accessed [October 10, 2024]. Available from: <https://www.fukushi.metro.tokyo.lg.jp/documents/d/fukushi/20240820_iryoukikanichiran-1-2>
22. **Japan Society of Obstetrics and Gynecology (JSOG).** Seishokuki no josei ni taisuru shakai-teki riyuu ni yoru ranshi no tōketsu ni kansuru ken [Statement on oocyte cryopreservation for social reasons in women of reproductive age]. Published October 2021. Accessed [November 10, 2024]. Available from: <https://www.jsog.or.jp/medical/865/>

**Islamic Countries**

1. **Shestak VA, Tsyplakova AD, Kholikov** IV. LEGAL SITUATION REGARDING ASSESSED REPRODUCTION TECHNOLOGIES IN MUSLIM COUNTRIES. Sib J Life Sci Agric. 2023 Feb 28;15(1):435–53.
2. **Al-Bar MA, Chamsi-Pasha H.** Contemporary Bioethics [Internet]. Cham: Springer International Publishing; 2015 [cited 2025 Apr 21]. Available from: http://link.springer.com/10.1007/978-3-319-18428-9

**India**

1. **Mahajan NK.** Optimising advice and approaches for elective fertility preservation. *Best Pract Res Clin Obstet Gynaecol*. 2025;99:102591. doi: 10.1016/j.bpobgyn.2025.102591
2. **Allahbadia GN.** Social egg freezing: developing countries are not exempt. *J Obstet Gynecol India*. 2016;66(4):213–217. doi:10.1007/s13224-016-0896-0.
3. **Bärnreuther S.** Racializing infertility: how South/Asian-ness has been constituted as an independent risk factor in infertility research and IVF practice. *Soc Sci Med*. 2021;280:114008. doi:10.1016/j.socscimed.2021.114008.
4. **Jindal UN.** Mid-life fertility: challenges and policy planning. *Indian J Med Res*. 2018;148(Suppl 1):S15–S26. doi:10.4103/ijmr.IJMR_40_18.
5. **Chatwal J, Bakshi R**, **Bakshi S, Tripathi N.** Social egg freezing: a gateway to fertility insurance. *Int J Reprod Contracept Obstet Gynecol.* 2023; 12(12):3588-3591. doi: <https://doi.org/10.18203/2320-1770.ijrcog20233639>.
6. **Desai S, Guharoy P, Mangoli V.** Nesting the Eggs on Another Day: Social Egg Freezing. *J Obstet Gynecol India*. 2025;75(3):192–8. doi: 10.1007/s13224-025-02157-x
7. **Rana R, Rana A, Mohanty S.** Oocyte Cryopreservation for Single Women: Where India Stands in 2021. *EC Gyneac.* 2021; 10(12): 24-26.
8. **Tyagi P, More A, Anjankar N, Dagwar P, Mahajan S.** A Case Report on Oocyte Cryopreservation in Assisted Reproductive Technique (ART) Empowering Parenthood. *J Pharm BioAllied Sci.* 2025;17(Suppl 1):S1023–25. doi: 10.4103/jpbs.jpbs_125_25
9. **Mishra R, Thakral S.** Legal subtleties of the Indian Assisted Reproductive Technology Act of 2021. *Natl Med J India*. 2025;37(5):272–274. doi: 10.25259/NMJI_785_2023
10. ***The Assisted Reproductive Technology (regulation) Act* *2021*** (India). Available from: <https://www.indiacode.nic.in/bitstream/123456789/17031/1/A2021-42%20.pdf>

**Iran**

1. **Akhondi MM, Ardakani ZB, Warmelink JC, Haghani S, Ranjbar F.** Knowledge and beliefs about oocyte cryopreservation for medical and social reasons in female students: a cross-sectional survey. *BMC Womens Health*. 2023;23(1):336. doi:10.1186/s12905-023-02535-6.
2. **Hafezi M, Zameni N, Nemati Aghamaleki SZ, Omani-Samani R, Vesali S.** Awareness and attitude toward oocyte cryopreservation for non-medical reasons: a study on women candidates for social egg freezing. J Psychosom Obstet Gynecol. 2022 Oct 2;43(4):532–40.

**Egypt**

1. **Chin AHB SSM**. Is social egg freezing (oocyte cryopreservation) for single women permissible in Islam? A perspective from Singapore.
2. **Chin AHB MS Ahmad MF.** Islamic Perspectives on Elective Ovarian Tissue Freezing by Single Women for Non-medical or Social Reasons.
3. **Mohamed N.** Effect of Educational Program on Knowledge and Attitude of Female Students’ Regarding Oocyte Cryopreservation. Tanta Sci Nurs J. 2023 May;29(2):264–80.
4. **Hasab Allah MF, Abdelnaem SA, Abuzaid ON.** Impact of educational guideline on nursing students' knowledge, beliefs and attitudes toward oocyte cryopreservation. Assiut Sci Nurs J. 2021;9(23):187–200. doi:10.21608/ASNJ.2021.91433.1223.
5. **Egypt’s Dar Al-Ifta** [Internet]. Petrie-Flom Center, Harvard Law School; 2019 Sep 23 [cited 2025 Jul 17]. Available from: <https://petrieflom.law.harvard.edu/2019/09/23/egg-freezing-permissible-in-islam-according-to-egypts-dar-al-ifta/>
6. **Al-Masir J M. Tajmīd al-buwayḍāt qabla al-ziwāj: dirāsa fiqhiyya muqārana** (Freezing eggs before marriage: A comparative jurisprudential study) [Internet]. Arabase. Al-Azhar University; 2019. Available from: [https://search.mandumah.com/Record/1540819](https://ddec1-0-en-ctp.trendmicro.com/wis/clicktime/v1/query?url=https%3a%2f%2fsearch.mandumah.com%2fRecord%2f1540819&umid=e796929b-3f32-43c8-a950-f4ba1cf6a396&rct=1752990180&auth=8d3ccd473d52f326e51c0f75cb32c9541898e5d5-d88bc4befcb162d49c0d09ae9c6e1a0f783b6b67)
7. **Abdullatif, Ramadan Abdulrahman F. Al-muqārabāt al-ṭibbiyya wa-l-ijtimāʿiyya al-thaqāfiyya li-taqniyati tajmīd al-buwayḍāt: dirāsa taḥlīliyya naqdiyya**(Medical and socio-cultural approaches of the Egg freezing “oocyte cryopreservation”: A critical analytical study) [Internet]. Arabase. 2022. Available from: [https://search.mandumah.com/Record/1297685](https://ddec1-0-en-ctp.trendmicro.com/wis/clicktime/v1/query?url=https%3a%2f%2fsearch.mandumah.com%2fRecord%2f1297685&umid=e796929b-3f32-43c8-a950-f4ba1cf6a396&rct=1752990180&auth=8d3ccd473d52f326e51c0f75cb32c9541898e5d5-ea91c070c53af0a97efdf8531e7b8cf796691046)
8. **Taya HHIH.** Tajmīd buwayḍāt al-fatāt al-ʿadhrāʾ wa atharuhu ʿala-l-bikāra: dirāsa fiqhiyya muqārana(Freezing the eggs of the virgin girl and its effect on the virginity: comparative jurisprudence study) [Internet]. Arabase. 2023. Available from: [https://search.mandumah.com/Record/1371051](https://ddec1-0-en-ctp.trendmicro.com/wis/clicktime/v1/query?url=https%3a%2f%2fsearch.mandumah.com%2fRecord%2f1371051&umid=e796929b-3f32-43c8-a950-f4ba1cf6a396&rct=1752990180&auth=8d3ccd473d52f326e51c0f75cb32c9541898e5d5-8f77db1ba4ec6cb102d222f9266d7bc76eb129c9)
9. **Al-Jarhi MMM.** Tajmīd al-buwayḍāt bayna al-fiqh al-islāmī wa al-taqaddum al-ṭibbī: ruʾya fiqhiyya ṭibbiyya muʿāṣara (Egg freezing between Islamic jurisprudence and medical development: contemporary medical jurisprudential perspective) [Internet]. Arabase. 2019. Available from: [https://search.mandumah.com/Record/1223424](https://ddec1-0-en-ctp.trendmicro.com/wis/clicktime/v1/query?url=https%3a%2f%2fsearch.mandumah.com%2fRecord%2f1223424&umid=e796929b-3f32-43c8-a950-f4ba1cf6a396&rct=1752990180&auth=8d3ccd473d52f326e51c0f75cb32c9541898e5d5-21a73e3a1bba673635e09b31f641f8e91b343108)
10. **Al-Koomi AMRAS, Eid A munim AS, Hamza MMH.** Mubarrirāt al-lujūʾ ilā inshāʾi bunūki tajmīd al-buwaydhāt (Justifications for resorting to establishing egg freezing banks (Self translated)) [Internet]. Arabase. 2022. Available from: [https://search.mandumah.com/Record/1309449](https://ddec1-0-en-ctp.trendmicro.com/wis/clicktime/v1/query?url=https%3a%2f%2fsearch.mandumah.com%2fRecord%2f1309449&umid=e796929b-3f32-43c8-a950-f4ba1cf6a396&rct=1752990180&auth=8d3ccd473d52f326e51c0f75cb32c9541898e5d5-ff172b33e75850860e27e67c2f50e8146b6cc760)
11. **Abdel Hafeez AKA**. Tajmīd al-baydhāt qabla al-ziwāj: ruʾya fiqhiyya fī dhawʾi al-mustajiddāt al-ṭibbiyya (Freezing eggs before marriage: A jurisprudential vision in the light of medical developments) [Internet]. Arabase. 2024. Available from: [https://search.mandumah.com/Record/1484161](https://ddec1-0-en-ctp.trendmicro.com/wis/clicktime/v1/query?url=https%3a%2f%2fsearch.mandumah.com%2fRecord%2f1484161&umid=e796929b-3f32-43c8-a950-f4ba1cf6a396&rct=1752990180&auth=8d3ccd473d52f326e51c0f75cb32c9541898e5d5-1649a95ed291087140c04042282cffe834d02497)
12. **Al-Kareem AGAG.** Al-ahkām al-fiqhiyya al-mutʿallaqa bi-l-buwaydhāt al-mulaqqaḥa al-mujammada: dirāsa fiqhiyya muqārana (The juristic provision of fertilized cryopreserved ocytes: A comparative juristic study) [Internet]. Arabase. 2019. Available from: [https://search.mandumah.com/Record/1032439](https://ddec1-0-en-ctp.trendmicro.com/wis/clicktime/v1/query?url=https%3a%2f%2fsearch.mandumah.com%2fRecord%2f1032439&umid=e796929b-3f32-43c8-a950-f4ba1cf6a396&rct=1752990180&auth=8d3ccd473d52f326e51c0f75cb32c9541898e5d5-81ad60d2d598d30da762b0a2a9d0b97659e989d5)
13. **Abdul Maqsood MGMM**. al-athar al-mutarattab a’lā talqīḥi buwaydhāt al-zawja bi-ghayri māi zawjihā: dirāsa fiqhiyya muqārana (The impact of fertilizing the wife’s egg with other than her husband’s semen: a comparative jurisprudential study) [Internet]. E-Marefa. 2024. Available from: [https://search.emarefa.net/ar/detail/BIM-1606271](https://ddec1-0-en-ctp.trendmicro.com/wis/clicktime/v1/query?url=https%3a%2f%2fsearch.emarefa.net%2far%2fdetail%2fBIM%2d1606271&umid=e796929b-3f32-43c8-a950-f4ba1cf6a396&rct=1752990180&auth=8d3ccd473d52f326e51c0f75cb32c9541898e5d5-b007af6dbadd45c9678c12b0eaddcd6a94f3a4e2)
14. **Al-Barbari BSA**. al-iltizāmāt al-nāshiʾa ʿan ʿaqdi tajmīd al-nuṭaf wa-l-buwaydhāt al-mukhaṣaba: dirāsa fiqhiyya muqārana(Obligations arising from the sperm and fertilized egg freezing contracts: A comparative jurisprudential study) [Internet]. 2024. Available from: [https://journals.ekb.eg/article_355242.html](https://ddec1-0-en-ctp.trendmicro.com/wis/clicktime/v1/query?url=https%3a%2f%2fjournals.ekb.eg%2farticle%5f355242.html&umid=69cfee05-b355-44fc-b566-79f247c38ab2&rct=1752993928&auth=8d3ccd473d52f326e51c0f75cb32c9541898e5d5-9d9c33f22d1b7cec662d03240001b5097f78fea2)

**Algeria**

1. Julud S. Al-talqīḥ al-iṣṭināʿī wa tajmīd al-buwaydhāt wa-l-ḥayawānāt al-manwiyya fi al-sharīʿa al-Islamiyya wa al-qānūn al-jazāirī (Artificial insemination and freezing oocytes and sperm in Islamic law and Algerian law) [Internet]. Arabase. 2022. Available from: [https://search.mandumah.com/Record/1201907](https://ddec1-0-en-ctp.trendmicro.com/wis/clicktime/v1/query?url=https%3a%2f%2fsearch.mandumah.com%2fRecord%2f1201907&umid=e796929b-3f32-43c8-a950-f4ba1cf6a396&rct=1752990180&auth=8d3ccd473d52f326e51c0f75cb32c9541898e5d5-6a202ff5f58a682e77c9773446ea07d1171d67fa)
